# Monitoring and risk analysis of residual pesticides drifted by unmanned aerial spraying

**DOI:** 10.1038/s41598-023-36822-w

**Published:** 2023-07-05

**Authors:** Chang Jo Kim, Xiu Yuan, Min Kim, Kee Sung Kyung, Hyun Ho Noh

**Affiliations:** 1 Residual Agrochemical Assessment Division, National Institute of Agricultural Sciences, Wanju, 55365 Korea; 2 Department of Environmental and Biological Chemistry, College of Agriculture, Life and Environment Science, Chungbuk National University, Cheongju, 28644 Korea

**Keywords:** Environmental sciences, Chemistry

## Abstract

This study aimed to investigate the residual characteristics of pesticides drifted by unmanned aerial spray according to buffer strip, windbreak, and morphological characteristics of non-target crops, suggest prevention for drift reduction, and finally conduct a risk analysis on pesticides exceeding the maximum residue limit (MRL) or uniform level (0.01 mg/kg) of the positive list system (PLS). Non-target crops were collected around the aerial sprayed area (paddy rice) in Boryeong, Seocheon, and Pyeongtaek after UAV spray. When pesticides were detected in more than three samples, Duncan’s multiple range test was performed. In cases where pesticides were detected in only two samples, an independent sample t-test was conducted (*p* < 0.05). The drift rate of pesticides tends to decrease by up to 100% as the buffer distance from aerial sprayed area increases or when a windbreak, such as maize, is present between two locations. Thus, the reduction of drifted pesticides could be effective if both factors were applied near the UAV spray area. Moreover, the residue of drifted pesticides was found to be the highest in leafy vegetables such as perilla leaves or leaf and stem vegetables such as Welsh onion, followed by fruit vegetables and cucurbits, owing to the morphological characteristics of crops. Therefore, selecting pulse or cereal such as soybean or maize as a farm product near the UAV spray area can be considered to minimize the drift. For pesticides that exceed the MRL or PLS uniform level, %acceptable dietary intake is 0–0.81% with no risk. Additionally, employing pesticides approved for both paddy rice and farm products in UAV spraying can effectively minimize instances where MRL or PLS are exceeded. Therefore, this study aims to provide farmers with effective guidelines for mitigating drift. Furthermore, we strive to promote stable and uninterrupted food production while facilitating the utilization of agricultural technologies such as UAV spraying to address labor shortages and ensure sustainable food security.

## Introduction

Pesticides application is considered a necessary procedure to protect agricultural products from harmful insects and diseases^1^, and total pesticide use has increased by approximately 50% in the 2020s compared to the 1990s^2^. However, concerns have been raised regarding the excessive use of pesticides and the risks they pose to both human health and the environment^3^. Furthermore, some countries are attempting to reduce the use of pesticides to achieve sustainable intensification (SI) in food production to meet the needs of a growing global population^4^.

However, in response to these concerns, the Agricultural Chemical Regulation Law^5^ and risk assessment^6^ have been implemented for the safe use of pesticides, as has been done in other developed countries^7^. Additionally, SI could be made feasible via technology, such as Internet of Things (IoT)^8^, big data^9^, artificial intelligence (AI)^10^, and unmanned aerial vehicles (UAVs)^11^ in agriculture. In particular, UAVs could prove to be an effective alternative solution to address labor shortages in agricultural work by enabling crop monitoring and pesticide spraying^12,13^, as the population of farmers has decreased while their average age has increased in some countries^14–16^.

Nevertheless, upon spraying pesticides with UAVs, the airborne pesticides could drift to non-target areas through the air^17^, resulting in unintentionally contaminating humans, plants, animals, and the environment^18^. To reduce pesticide drift, some factors have been studied, including (1) meteorological conditions^19^ such as wind direction and speed^20^, humidity, and temperature^21^; (2) UAV spray conditions such as spray pressure^22^, flight height^23^, and flight speed^24^; (3) UAV components such as rotor^25^ and nozzle^26,27^; and (4) physical properties of spray solutions according to adjuvant^28^ and formulation^29^.

However, in the context of aerial spraying conducted over paddy rice fields in diverse topographies during a specific period in Korea, the factors contributing to drift, including meteorological conditions, UAV spray conditions, UAV components, types of UAVs (multicopter or helicopter), and physical properties of the spray solution^30^, were not identified. Furthermore, it is important to note that drifted pesticides have the potential to impact individuals residing in proximity to farming areas^31^ and pose a risk of drifting onto non-target crops, considering that UAVs primarily apply pesticides onto paddy fields^32^. In such a case, the residue of pesticides in these crops would exceed the maximum residue limits (MRL) or positive list system (PLS) uniform standard (0.01 mg/kg), which could pose a risk to human health if such contaminated crops are ingested.

However, buffer strips^33^, windbreaks^34^, and morphological characteristics^35^ can potentially impact the residue of drifted pesticides and should be investigated following UAV spraying. Furthermore, although drift mitigation measures have shown some reduction in pesticide contamination over time, significant risks to human health and the environment still persist^36^. Therefore, it is necessary to monitor drifted pesticides considering these three factors and conduct risk analysis after UAV pesticide application to ensure effective prevention. Hence, this study aims to achieve the following objectives: (1) investigate the residual characteristics of pesticides that have drifted onto non-target crops surrounding the aerial sprayed area (paddy rice), taking into account three factors, that is, buffer strip, windbreak, and morphological characteristics of non-target crops; (2) implement preventive measures to reduce pesticide drift by monitoring various non-target crops near paddy rice in three regions (Boryeong, Seocheon, and Pyeongtaek) in the Republic of Korea; and (3) conduct a risk analysis to assess the risk posed by drifted pesticides in non-target crops, utilizing the residue levels of pesticides exceeding the standard MRL or PLS (0.01 mg/kg).

## Materials and methods

### Target pesticide and sample

Dinotefuran + etofenprox 13(5 + 3)% micro-emulsion and azoxystrobin + propiconaozle 18.71(7.01 + 11.7)% suspo-emulsion were sprayed in Boryeong-si, and dinotefuran + etofenprox 13(5 + 3)% and azoxystrobin + hexaconazole 13(12 + 1)% suspension concentrate (SC) were sprayed in Seocheon-gun, Chungcheongnam-do, Republic of Korea. Additionally, azoxystrobin + ferimzone 21.5(6.5 + 15)% SC and chlorantraniliprole + clothianidin 4.7(2.7 + 2)% SC were sprayed in Pyeongtaek-si, Gyeonggi-do, Republic of Korea. The test pesticides used in this study include insecticides such as chlorantraniliprole (diamide), clothianidin (neonicotinoid), dinotefuran (neonicotinoid), and etofenprox (pyrethroid), as well as fungicides such as azoxystrobin (strobilurin), ferimzone (pyrimidine), hexaconazole (triazole), and propiconazole (triazole). The target pesticides in each location were sprayed onto paddy rice with UAVs, following land registration maps that confirmed the aerial spray area. The land registration maps were obtained from the National Agricultural Cooperative Federation, and then crops around aerial sprayed area were collected with optional information, such as the distance (in meters) and windbreak between the collected crops and the aerial sprayed area, to determine the residual characteristics of the drifted pesticides.

The collected samples include chili pepper (*Capsicum annuum*), squash (*Cucurbita pepo*), squash leaves, dureup (*Aralia cordata*), perilla leaves (*Perilla frutescens*), Welsh onion (*Allium fistulosum*), tomato (*Solanum lycopersicum*), cucumber (*Cucumis sativus*), eggplant (*Solanum melongena*), peach (*Cucumis sativus*), apple (*Malus pumila*), grape (*Vitis vinifera*), pear (*Pyrus pyrifolia*), soybean (*Glycine max*), soybean leaves, maize (*Zea mays*), white-flowered gourd (*Lagenaria siceraria*), and white-flowered gourd leaves with inedible parts, such as maize leaves and sesame leaves (*Sesamum indicum*), to evaluate the residual characteristics of drifted pesticides (Supplementary Table S1). Collected samples were stored below − 20 ℃ immediately after chopping and blending with dry ice^37^.

### Approval statement

The samples were collected for monitoring pesticide drift by UAV spray with the approval of the farmers. Additionally, all methods, from collection of samples to analysis of residual pesticides, were performed in accordance with the relevant guidelines and regulations of the National Institute of Agricultural Sciences (NAS) and Ministry of Government Legislation (MOLEG).

### Reagent and instrument

Reference materials (RM) for etofenprox (purity > 99.0%), hexaconazole (purity > 98.7%), and propiconazole (purity > 98.5%) were obtained from Dr. Ehrenstrofer GmbH (Augsburg, Germany) and were weighted with precision balance (New Jersey, US) to prepare stock solution. Azoxystrobin, clothianidin, chlorantraniliprole, dinotefuran, (*E*)-ferimzone, (*Z*)-ferimzone, and 1000 mg/L stock solution were obtained from Accustandard (New Haven, USA). LiChrosolv-grade acetonitrile and methanol were secured from Merk (Darmstadt, Germany). QuEChER EN packet (Cat No. 5982-5650) and dispersive-SPE (Cat No. 5982-5021) were obtained from Agilent Technologies (California, US). Deionized water was used along with Autwomatic Plus 1 + 2 from Waaserlab (de Navarra, Spain). Formic acid (purity > 98.0%) was secured from Merk (Darmstadt, Germany). The extract machine used was the 2010 Geno/Grinder from SPEX SamplePrep (Metuchen, US), and the vortex mixer was Vortex-Genie 2 from Scientific industry (New York, US). Finally, the centrifuge was from Hanil Science (Incheon, Korea).

### Stock and working solution

To prepare 1,000 mg/L of stock solutions of pesticides, each RM was weighted with precision balance, considering the purities of the RM. Thus, 20.26 mg of etofenprox, 20.20 mg of hexaconazole, and 20.30 mg of propiconazole were dissolved in 20 mL of methanol. Each stock solution was diluted with acetonitrile to concentrations ranging from 0.005 to 100 mg/L. These concentrations were used to plot the regression curve and conduct the recovery test.

### Sample preparation

The sample preparation was conducted according to the QuEChERS method^38^. The samples (10 g) were placed in a 50-mL conical centrifuge tube (FalcornTM, US) and shaken with acetonitrile (10 mL) for 5 min at 1300 rpm. The sample was then shaken again under the same conditions with magnesium sulfate (4 g), sodium chloride (1 g), trisodium citrate dihydrate (1 g), and disodium hydrogencitrate sesquihydrate (0.5 g) (QuEChERS EN extraction packet). The mixture was centrifuged for 5 min at 3500 rpm, and the supernatant (1 mL) was added in a dispersive Solid-Phase Extraction (d-SPE) tube containing magnesium sulfate (150 mg) and primary secondary amine (PSA) (25 mg) for clean-up. The tube was then vortexed for 30 s. The purified solution was filtered using a syringe filter (PTFE, 13 mm, 0.22 µm) after centrifuging for 5 min at 12,000 rpm. The supernatant was then mixed in a 50:50 (v/v) proportion with acetonitrile to create a matrix-matched sample, which was analyzed with LC–MS/MS according to Supplementary Table S2^39^. For soybean, the sample was analyzed directly after matrix matching without performing the purification procedure.

### Verification of analysis method

The limit of quantitation (LOQ) was determined by setting a signal-to-noise ratio of over 10 in a matrix-matched standard considering the PLS uniform standard (0.01 mg/kg)^40^. A regression curve for calibration was plotted by analyzing more than five matrix-matched standards and comparing the concentration and intensity of the peaks to evaluate the correlation coefficient (*r*^2^) according to SANTE/11312/2021^41^. The recovery test was conducted with apple, wakegi onion, perilla leaves, and soybean as representative crops from commodity groups of collected crops^42^. The accuracy and repetition of the recovery test were evaluated using three fortification levels of LOQ, 10 LOQ, and 50 LOQ with recovery (%) and relative standard deviation (RSD) according to performance criteria for analysis of pesticide^43^.

### Decision on drifted pesticides

To understand the drift of pesticides through UAV spraying, a wealth of information is required, encompassing meteorological conditions, UAV types, spray conditions, UAV components, and physical properties of the spray solution that can influence drift. However, in this study, pesticides were simultaneously sprayed onto paddy rice in various topographies using two types of UAVs within a specific timeframe. Consequently, it was not feasible to capture all the details during each spraying event. Additionally, the presence of residual pesticides in non-target crops may not be solely attributed to UAV drift but could also be attributed to farmer practices. Hence, we explore other factors that could influence drift and are applicable for investigation even after UAV spraying.

The residue of drifted pesticides is influenced by plant morphology^44^, buffer strips^45^, and windbreaks^46^. Therefore, we examined these factors when collecting non-target crops. To understand the characteristics of residual pesticides, we analyze the pesticide residues based on three factors: buffer strip, crop morphology, and windbreak. Furthermore, we investigate whether the aerially sprayed pesticides are commonly detected and registered in the harvested crops.

### Risk analysis

A risk analysis was conducted using estimated daily intake (EDI) and % acceptable daily intake (%ADI) in cases where the residue of pesticides in crop around aerial sprayed area exceed MRL or PLS uniform standard (0.01 mg/kg), specifically for crops with an established ADI^47^. To calculate EDI and %ADI, food consumption (g/day) was determined from the “National Food & Nutrition Statistics”^48^. Moreover, the average weight of an adult in Korea, which is 66.55 kg, was established based on the “National Health Screening Statistical Yearbook” (Eqs. 1 and 2)^49^.

1 $${\text{EDI }} = \frac{{\left\{ {{\text{Residual}}\;{\text{concentration}} \left( {{\text{mg}}/{\text{kg}}} \right) \times {\text{daily}}\;{\text{food}}\;{\text{intake}} \left( {{\text{g}}\;{\text{bw}}/{\text{day}}} \right)} \right\}}}{1000}$$

2 $$\% {\text{ADI}} = \left[ {\frac{{\left\{ {{\text{EDI}}/66.55\;{\text{kg}} \left( {{\text{Average}}\;{\text{body}}\;{\text{weight}}\,{\text{Korean}}} \right)} \right\}}}{{{\text{ADI}}}}} \right] \times 100$$

### Statistical analysis

To plot a calibration regression curve and assess the correlation coefficient, Microsoft Excel (USA) was utilized. The residual characteristics of the collected samples with drifted pesticides were analyzed using Statistical Package for the Social Sciences (SPSS) software (Ver. 26, IBM Corporation, USA). In cases where pesticides were detected in only two samples at a specific site, an independent sample t-test was employed for residue analysis. In cases where pesticides were detected in more than three samples, a one-way analysis of variance (ANOVA) was conducted, followed by Duncan's multiple range test (DMRT) with a significance level of *p* < 0.05.

## Results and discussion

### Verification of analysis

The LOQ of all pesticides, including azoxystrobin, chlorantraniliprole, clothianidin, dinotefuran, etofenprox, ferimzone, hexaconazole, and propiconazole, was 0.01 mg/kg. The linearity of all matrix-matched standards was high, with a correlation coefficient higher than 0.99. The recoveries of target pesticides in representative crops ranged from 72.3 to 116.6% (with a relative standard deviation (RSD) of 0.2–10.7%). Thus, it can be concluded that the sample preparation and LC–MS/MS conditions were appropriate for analyzing the pesticides (Supplementary Table S3).

### Analysis result of residual pesticide

#### Decision on drifted pesticides

The investigation of pesticide residue in crops considering various factors including morphology of the crop, buffer distance, and windbreaks (Tables 1, 2 and 3) suggests that the target pesticides sprayed by UAVs did not drift in 13 out of 39 locations where samples were collected (location nos. 1, 3, 6-1, 6-2, 6-3, 7, 13, 18, 19, 20, 22, 26, 27) at a significance level of *p* < 0.05. Target pesticides were not detected in seven of these locations (location nos. 6-3, 7, 13, 18, 22, 26, 27). In the remaining locations (location nos. 1, 3, 6-1, 6-2, 19, 20), although a maximum of two pesticides were detected, the residue of pesticides did not decrease as distance from the aerial sprayed area increased (*p* < 0.05). Therefore, the residual pesticides in non-target crops were probably already present before the UAV spraying.

**Table 1:** Residual concentration of pesticides in the crops surrounding paddy rice sprayed with UAV in Boryeong.

Location no	Crop	Distance from spray area (m)	Windbreak (height, m)	Residue (mean ± SD, mg/kg)			
				Azoxystrobin	Dinotefuran	Etofenprox	Propiconazole
1^a^	Soybean leaves	9	–	< LOQ	< LOQ	0.59 ± 0.02 e	< LOQ
		11	–	< LOQ	< LOQ	1.58 ± 0.07 d	< LOQ
		13	–	< LOQ	< LOQ	2.54 ± 0.09 b	< LOQ
		15	–	< LOQ	< LOQ	3.01 ± 0.03 a	< LOQ
		17	–	< LOQ	< LOQ	2.12 ± 0.13 c	< LOQ
	Peach	3.5	–	< LOQ	< LOQ	2.88 ± 0.20 a	< LOQ
2	Squash leaves	1	–	0.01 ± 0.00	0.02 ± 0.00 *	0.01 ± 0.00	0.01 ± 0.00
		8	–	< LOQ	0.03 ± 0.00 *	< LOQ	< LOQ
3	Chili pepper^c^	3.5	–	< LOQ	0.14 ± 0.00 a	< LOQ	< LOQ
		5.5		< LOQ	0.02 ± 0.00 c	< LOQ	< LOQ
		7.5		< LOQ	0.13 ± 0.01 b	< LOQ	< LOQ
4	Chili pepper	6	–	< LOQ	0.05 ± 0.00 c	0.03 ± 0.00	0.03 ± 0.00
		8	–	< LOQ	0.10 ± 0.00 b	< LOQ	< LOQ
		10	–	< LOQ	0.16 ± 0.01 c	< LOQ	< LOQ
5	Chili pepper	5	–	0.12 ± 0.01 c	< LOQ	0.01 ± 0.00	< LOQ
		7	–	0.18 ± 0.00 a	< LOQ	< LOQ	< LOQ
		9	–	0.19 ± 0.01 a	< LOQ	< LOQ	< LOQ
		18	–	0.16 ± 0.01 b	< LOQ	< LOQ	< LOQ
6-1	Chili pepper	4	Structure (2)	0.03 ± 0.00	< LOQ	< LOQ	< LOQ
6-2	Maize	9	–	< LOQ	< LOQ	< LOQ	< LOQ
	Maize leaves			0.02 ± 0.00 c	< LOQ	< LOQ	< LOQ
	Chili pepper	9	Maize (1.5)	0.04 ± 0.00 a	0.02 ± 0.00 *	< LOQ	< LOQ
		14		0.03 ± 0.00 b	0.04 ± 0.00 *	< LOQ	< LOQ
	Maize	19		< LOQ	< LOQ	< LOQ	< LOQ
	Maize leaves			< LOQ	< LOQ	< LOQ	< LOQ
6-3	Eggplant	9	–	< LOQ	< LOQ	< LOQ	< LOQ
6-4	Maize	3	Maize (1.5)	< LOQ	< LOQ	< LOQ	< LOQ
	Maize leaves			< LOQ	< LOQ	0.04 ± 0.00 b	0.02 ± 0.00 b
	Squash leaves	3.5	Maize (1.5)	< LOQ	< LOQ	0.13 ± 0.01 a	0.04 ± 0.00 a
		10		< LOQ	< LOQ	0.04 ± 0.00 b	0.02 ± 0.00 b
7	Chili pepper	2	–	< LOQ	< LOQ	< LOQ	< LOQ
	Squash	3		< LOQ	< LOQ	< LOQ	< LOQ
	Chili pepper^d^	5		< LOQ	< LOQ	< LOQ	< LOQ
		7.5	Pumpkin leaves (1.5)	< LOQ	< LOQ	< LOQ	< LOQ
		8	–	< LOQ	< LOQ	< LOQ	< LOQ
8	Dureup	5	–	< LOQ	< LOQ	< LOQ	0.02 ± 0.00
		10	–	< LOQ	< LOQ	< LOQ	< LOQ
		22	–	< LOQ	< LOQ	< LOQ	< LOQ
9	Perilla leaves	1	–	10.72 ± 0.31 *	< LOQ	7.00 ± 0.16 *	13.31 ± 0.37 *
	Chili pepper	2	–	0.08 ± 0.00 *	< LOQ	0.13 ± 0.01 *	0.15 ± 0.02 *
10	Sesame leaves	1	–	1.31 ± 0.00	< LOQ	1.19 ± 0.01 *	1.36 ± 0.05
	Chili pepper	2	Sesame leaves (1.5)	< LOQ	< LOQ	0.06 ± 0.01 *	< LOQ
11^b^	Chili pepper	2	–	0.01 ± 0.00 b	0.03 ± 0.00 c	0.03 ± 0.00 b	0.03 ± 0.00 b
		2	–	0.07 ± 0.01 a	0.06 ± 0.00 b	0.11 ± 0.01 a	0.14 ± 0.01 a
		2	–	0.01 ± 0.01 b	0.02 ± 0.00 d	0.02 ± 0.01 b	0.03 ± 0.01 b
		7	–	< LOQ	0.17 ± 0.00 a	< LOQ	< LOQ
		7	–	< LOQ	0.18 ± 0.00 a	< LOQ	< LOQ
12	White-flowered gourd	0	–	< LOQ	< LOQ	0.04 ± 0.00 c	0.03 ± 0.00 c
		2		< LOQ	< LOQ	< LOQ	< LOQ
		6	Maize (2)	< LOQ	< LOQ	< LOQ	< LOQ
		11		< LOQ	< LOQ	< LOQ	< LOQ
	White-flowered gourd leaves	0	–	2.08 ± 0.11 *	< LOQ	3.51 ± 0.18 a	3.99 ± 0.08 a
		2		0.02 ± 0.00 *	< LOQ	0.53 ± 0.00 b	0.41 ± 0.02 b
		6	Maize (2)	< LOQ	< LOQ	0.03 ± 0.00 c	0.01 ± 0.00 c
		11		< LOQ	< LOQ	< LOQ	< LOQ
	Maize	5		< LOQ	< LOQ	< LOQ	< LOQ
		10		< LOQ	< LOQ	< LOQ	< LOQ
	Maize leaves	5		< LOQ	< LOQ	0.03 ± 0.00 c	0.01 ± 0.00 c
		10		< LOQ	< LOQ	< LOQ	< LOQ
13	Chili pepper	5	–	< LOQ	< LOQ	< LOQ	< LOQ
			Maize (1.8)	< LOQ	< LOQ	< LOQ	< LOQ
14	Chili pepper	3	–	0.18 ± 0.01 c	< LOQ	0.01 ± 0.00	0.02 ± 0.00
		5	–	2.87 ± 0.21 a	< LOQ	< LOQ	< LOQ
		7	–	2.11 ± 0.11 b	< LOQ	< LOQ	< LOQ
15	Welsh onion	2.5	–	0.43 ± 0.03 *	< LOQ	0.36 ± 0.02 *	0.61 ± 0.03 *
		5	–	0.04 ± 0.00 *	< LOQ	0.02 ± 0.00 *	0.05 ± 0.00 *
	Chili pepper	5.5	–	< LOQ	< LOQ	< LOQ	< LOQ
		9.5	–	< LOQ	< LOQ	< LOQ	< LOQ
		13.5	–	< LOQ	< LOQ	< LOQ	< LOQ
16	Welsh onion	1	–	0.05 ± 0.00 d	< LOQ	0.04 ± 0.00 a	0.07 ± 0.00 a
			Tree (3.5)	0.04 ± 0.00 d	< LOQ	0.01 ± 0.00 b	0.02 ± 0.00 b
	Chili pepper	4	–	1.83 ± 0.05 c	< LOQ	< LOQ	0.02 ± 0.00 c
		6	–	2.50 ± 0.03 b	< LOQ	< LOQ	< LOQ
		8	–	4.85 ± 0.17 a	< LOQ	< LOQ	< LOQ
17	Apple	0	–	0.03 ± 0.00 b	< LOQ	0.06 ± 0.00 c	0.06 ± 0.00 c
	Welsh onion	1	–	0.24 ± 0.00 a	< LOQ	0.19 ± 0.01 a	0.47 ± 0.00 a
	Chili pepper	3	Tree (5)	< LOQ	< LOQ	< LOQ	0.02 ± 0.00 d
			–	0.02 ± 0.00 c	< LOQ	0.11 ± 0.01 b	0.14 ± 0.00 b

^a^It was considered that the pesticide was already sprayed before UAV spraying took place.

^b^Location was surrounded by UAV sprayed area.

^c^It was grown in vinyl house with a side window opened by 0.8 m.

^d^It was grown in vinyl house.

*There were significant differences at the *p* < 0.05 level by t-test.

No significant difference exists within the same location if the same small letter appears in the same column (*p* < 0.05).

**Table 2:** Residual concentration of pesticides in the crops surrounding paddy rice sprayed with UAV in Seocheon.

Location no	Crop	Distance from spray area (m)	Wind break (height, m)	Residue (mean ± SD, mg/kg)			
				Azoxystrobin	Dinotefuran	Etofenprox	Hexaconazole
18^a^	Chili pepper	10.5	–	< LOQ	< LOQ	< LOQ	< LOQ
		12.5	–	< LOQ	< LOQ	< LOQ	< LOQ
		14.5	–	< LOQ	< LOQ	< LOQ	< LOQ
		20.5	–	< LOQ	< LOQ	< LOQ	< LOQ
19	Chili pepper	6	–	< LOQ	0.04 ± 0.00 b	< LOQ	< LOQ
		7.5	–	< LOQ	0.03 ± 0.00 c	< LOQ	< LOQ
		9	–	< LOQ	0.03 ± 0.00 d	< LOQ	< LOQ
		10.5	–	< LOQ	0.05 ± 0.00 a	< LOQ	< LOQ
		12	–	< LOQ	0.04 ± 0.00 b	< LOQ	< LOQ
20	Grape	0	–	< LOQ	0.06 ± 0.00*	< LOQ	< LOQ
		3^c^		< LOQ	0.07 ± 0.00*	< LOQ	< LOQ
21^b^	Soybean	1.5	–	0.02 ± 0.00 c	0.02 ± 0.00*	0.02 ± 0.00*	< LOQ
	Soybean leaves			2.67 ± 0.17 b	0.92 ± 0.01*	1.17 ± 0.05*	0.08 ± 0.00
	Soybean	5.5	Soybean (2)	0.03 ± 0.00 c	< LOQ	< LOQ	< LOQ
	Soybean leaves			13.22 ± 0.89 a	< LOQ	< LOQ	< LOQ
22	Peach	4.5	–	< LOQ	< LOQ	< LOQ	< LOQ
		7.5	–	< LOQ	< LOQ	< LOQ	< LOQ
23	Peach	0	–	< LOQ	0.02 ± 0.00	0.04 ± 0.01	< LOQ
	Pear	1	–	< LOQ	< LOQ	< LOQ	< LOQ
		3	–	< LOQ	< LOQ	< LOQ	< LOQ
24	Pear	2.5	–	< LOQ	< LOQ	< LOQ	< LOQ
	Apple		–	< LOQ	< LOQ	< LOQ	< LOQ
	Peach	3	–	< LOQ	0.03 ± 0.00	0.03 ± 0.00	< LOQ
25	Soybean leaves	0	–	2.50 ± 0.06 a	2.08 ± 0.08 a	8.84 ± 0.38 a	0.33 ± 0.02 a
		1.5	–	0.45 ± 0.02 b	0.59 ± 0.03 b	3.68 ± 0.18 b	0.12 ± 0.00 b
	Maize	3.5	–	< LOQ	< LOQ	< LOQ	< LOQ
26	Peach	4	–	< LOQ	< LOQ	< LOQ	< LOQ

^a^It was considered that the pesticide was already sprayed before UAV spraying took place.

^b^It was grown in vinyl house with a side window opened by 1 m.

^c^It was grown in vinyl house.

*There were significant differences at *p* < 0.05 level by t-test.

No significant difference exists within the same location if the same small letter appears in the same column (*p* < 0.05).

**Table 3:** Residual concentration of pesticides in the crops surrounding paddy rice sprayed with UAV in Pyeongtaek.

Location no	Crop	Distance from spray area (m)	Wind break (height, m)	Residue (mean ± SD, mg/kg)			
				Azoxystrobin	Clothianidin	Chlorantraniliprole	Ferimzone
27^a^	Chili pepper	2.5	–	< LOQ	< LOQ	< LOQ	< LOQ
	Eggplant		–	< LOQ	< LOQ	< LOQ	< LOQ
	Chili pepper	6	–	< LOQ	< LOQ	< LOQ	< LOQ
	Eggplant		–	< LOQ	< LOQ	< LOQ	< LOQ
28	Soybean leaves	1	–	0.02 ± 0.00	< LOQ	< LOQ	0.03 ± 0.00
	Welsh onion		–	< LOQ	< LOQ	< LOQ	< LOQ
29	Squash leaves	0.5	–	0.01 ± 0.00	< LOQ	< LOQ	0.03 ± 0.00
	Tomato	4.5	Voluble stem of pumpkin (1.5)	< LOQ	< LOQ	< LOQ	< LOQ
	Cucumber			< LOQ	< LOQ	< LOQ	< LOQ
	Eggplant			< LOQ	< LOQ	< LOQ	< LOQ
	Chili pepper			< LOQ	< LOQ	< LOQ	< LOQ
	Apple	13		< LOQ	< LOQ	< LOQ	< LOQ
30	Soybean leaves	1	–	1.86 ± 0.03 b	0.27 ± 0.01 a	0.53 ± 0.01 a	4.45 ± 0.24 a
	Chili pepper		–	0.11 ± 0.00 d	0.02 ± 0.00 e	0.03 ± 0.00 f.	0.30 ± 0.01 d
	Eggplant		–	< LOQ	< LOQ	< LOQ	< LOQ
	Welsh onion		–	0.41 ± 0.02 c	0.06 ± 0.00 c	0.12 ± 0.00 c	0.94 ± 0.02 c
	Perilla leaves		–	6.14 ± 0.08 a	0.24 ± 0.02 b	0.38 ± 0.02 b	3.35 ± 0.10 b
	Soybean leaves	6	–	0.38 ± 0.01 c	0.05 ± 0.00 cd	0.10 ± 0.00 d	0.88 ± 0.02 c
			Maize (1.5)	0.13 ± 0.01 d	0.04 ± 0.01 d	0.07 ± 0.01 e	0.39 ± 0.04 d
31	Welsh onion	3.5	–	0.12 ± 0.01	0.01 ± 0.00	0.03 ± 0.00	0.18 ± 0.02
32	Chili pepper	3	–	0.18 ± 0.01 a	0.24 ± 0.01 b	0.06 ± 0.00 a	0.47 ± 0.03 a
		4	–	0.09 ± 0.00 b	0.30 ± 0.01 a	0.03 ± 0.00 b	0.19 ± 0.01 b
		5	–	0.03 ± 0.00 c	0.18 ± 0.00 c	0.01 ± 0.00 c	0.08 ± 0.00 c
		6	–	0.04 ± 0.00 c	0.31 ± 0.01 a	0.01 ± 0.00 d	0.08 ± 0.00 c
		7	–	< LOQ	0.23 ± 0.01 b	< LOQ	0.01 ± 0.00 d
33	Peach	2.5	–	< LOQ	0.05 ± 0.00*	0.06 ± 0.00*	< LOQ
		9	–	< LOQ	0.04 ± 0.00*	0.04 ± 0.00*	< LOQ
34	Welsh onion	1	–	0.24 ± 0.00 a	0.04 ± 0.00*	0.09 ± 0.01*	0.51 ± 0.00 a
	Chili pepper		–	0.11 ± 0.01 b	0.01 ± 0.00*	0.03 ± 0.00*	0.21 ± 0.02 b
	Eggplant		–	0.03 ± 0.00 c	< LOQ	< LOQ	0.05 ± 0.00 c
35	Chili pepper	2.3	–	0.01 ± 0.00	< LOQ	< LOQ	0.04 ± 0.00
		3.6	–	< LOQ	< LOQ	< LOQ	< LOQ
		4.9	–	< LOQ	< LOQ	< LOQ	< LOQ
36	Welsh onion	1.5	–	0.13 ± 0.02 b	0.02 ± 0.00*	0.05 ± 0.01*	0.28 ± 0.03 b
	Eggplant^b^		–	0.24 ± 0.01 a	0.04 ± 0.00*	0.07 ± 0.00*	0.46 ± 0.02 a
	Tomato		–	0.02 ± 0.00 c	< LOQ	< LOQ	0.03 ± 0.00 c

^a^It was considered that the pesticide was already sprayed before UAV spraying took place.

^b^Location was an interjection of two sides of UAV sprayed area.

*There were significant differences at *p* < 0.05 level by t-test.

No significant difference exists within the same location if the same small letter appears in the same column (*p* < 0.05).

In three locations (location nos. 1, 14, and 16), the residues of both etofenprox in peach and azoxystrobin in chili pepper exceeded the MRL (Tables 1, 2 and 3). However, the other pesticides sprayed by UAVs were not detected, and there was no evidence of drifted pesticides, as the residue of two pesticides did not decrease as the distance increased from the sprayed area^32^. Furthermore, azoxystrobin is commonly used in farm products such as chili pepper^50^, and etofenprox is frequently detected in both herbal fruits and stalk and stem vegetables^51,52^. Therefore, it can be concluded that the five cases that exceeded the MRL were not due to the drift of airborne pesticides but rather the presence of pesticides that were already sprayed before the UAV spraying.

In total, the residue of pesticides exceeded the PLS uniform standard in 31 cases across 15 locations (location nos. 2, 6-4, 10, 12, 21, 25, 28, 29, 30, 31, 32, 33, 34, 35, 36). These include one case of etofenprox in white-flowered gourd, five cases of propiconazole (three cases of squash leaves and a case of dureup and white-flowered gourd), 21 cases of ferimzone (four cases of soybean leaves, eight cases of chili pepper, four cases of Welsh onion, two cases of eggplant, and one case each of squash leaves, perilla leaves, and tomato), and four cases of dinotefuran in soybean leaves. It was inferred that the pesticides that exceeded the PLS uniform standards were drifted by UAV spray, as all the cases showed uniform residual tendency of pesticides according to distance from the sprayed area, windbreak, and morphology of crops^32^, and the pesticides sprayed by UAV were commonly detected. It is recommended that pesticides for UAV spray be chosen in the case of both rice and farm products, considering ferimzone, which is only applied to rice, and the 68% of total cases that exceeded PLS.

#### Drift characteristics according to buffer strip

Airborne pesticides were found to decrease as the distance from the aerial sprayed area increased, with 0–100% drift reduction (location nos. 2, 4, 5, 6-4, 8, 9, 11, 12, 14, 15, 21, 25, 29, 30, 32, 33, 35). This trend is consistent with the results of wind tunnel and field tests, which also showed that the amount of airborne pesticides decreased with increasing distance^22,53^. The samples were collected from distances ranging from 0 to 22 m from the aerial sprayed area, with an average distance of 5.7 m. It appears that crops were grown around aerial sprayed area without a uniform buffer strip. Therefore, it would be appropriate to establish a uniform buffer strip around the aerial sprayed area^34^. However, target pesticides were detected in non-target fruits and leafy vegetables, such as squash leaves, white-flowered gourd leaves, and maize leaves, beyond 5.7 m from the sprayed area (Tables 1, 2 and 3). Moreover, wind speeds have been identified as a factor influencing drift deposition^54^, and it has been advised to establish a buffer strip ranging from 25 to 300 m in the case of herbicide spraying^34^. However, the effectiveness of a buffer strip in reducing pesticide drift can vary, resulting in inconsistent levels of drift reduction ranging from 0 to 100%. This variability is attributed to the influence of other factors, including meteorological conditions, morphological characteristics of non-target crops, and UAV spray conditions. Consequently, relying solely on the implementation of a buffer strip may prove insufficient in preventing drift.

#### Drift characteristics according to crop morphology

The residue of airborne pesticides in non-target samples varies according to the morphological characteristics of the collected samples (location nos. 6-4, 9, 12, 17, 21, 23, 24, 25, 28, 30, 34, 36). Analysis results show that pesticide residue was higher in leafy or stalk and stem vegetables than fruiting vegetables other than cucurbits, followed by cucurbits (*p* < 0.05). Soybean and maize appear to be less susceptible to drifted pesticides because of their outer layer, and the residue of pesticides was less than in other crops (Tables 1, 2 and 3). This tendency is similar to earlier reports stating that the residue of carbamates pesticide was the lowest in cereal and pulses^55^ and that residues of pesticides were higher in leafy vegetables than in leaves of root and bulb vegetables, including pulses with pods, fruits, pulses and cereal grains, and root and bulb vegetables^44^.

In addition, peach, perilla leaves, and squash leaves with glandular hair tended to have higher residues of pesticides (*p* < 0.05). The deposition of droplets was affected by components such as microstructure, wax, stomata, and hair on the surface of leaves^56^. The retention of droplets improves in leaves with longer even hairs or rougher surface^57^. Additionally, four types of leaves, namely cocklebur, morning glory, velvet leaf, and coffee senna, showed 99, 77, 65, and 55% of deposition efficiencies, respectively, when sprayed with 140 µm of droplets^35^. Similarly, needle-like leaves had two to four times higher deposition than broad leaves^58^; thus, it was concluded that the amount of deposit of airborne pesticides could differ according to the morphological characteristics of crops. Consequently, it is recommended to grow crops less susceptible to retention of airborne pesticides around aerial sprayed area to prevent unintentional contamination.

Given the early- or mid-growth stages of the collected samples, it was not an appropriate time to assess the safety of pesticides. Furthermore, the residue of pesticides tends to dissipate or degrade over time due to the growth of agricultural products, which is influenced by meteorological conditions such as radiation, temperature, humidity, and rainfall, as well as the physio-chemical properties of the pesticides^59^. Consequently, it is likely that the residue of drifted pesticides in farm products will decrease by the time of harvest. The following section describes the residual patterns of drifted pesticides, assuming that they were caused by drift.

#### Drift characteristics according to windbreak

The analysis of residual pesticides indicates that the residues of pesticides were lower when windbreaks were between non-target crop and aerial sprayed area (location nos. 10, 16, 17, 21, 25, 30, *p* < 0.05). Notably, the analysis reveals a reduction of 30–100% in the residue of drifted pesticides, even when the same crops were collected from the same distance from the aerial sprayed area (location nos. 16, 17, 30, *p* < 0.05). The degree of drift varied based on several factors, such as structural parameters (porosity, length, width, and height) and climatic factors, among others^34,60–62^. For instance, the drift of airborne pesticides by a boom sprayer or UAV was reduced when maize was used as a windbreak^34,46^. Thus, proper placement of windbreaks tailored to the topography of each location can prevent unintentional contamination (Supplementary Figs. S1–S3).

### Risk analysis

The result of the risk analysis involving 36 cases that exceeded the MRL or PLS uniform standard showed that the %ADI ranged from 0.00 to 0.81% (Table 4); this shows that there was no risk^63^. Considering that processing treatments, including washing, peeling, heat treatments, and drying, can effectively eliminate residual pesticides^64^, the likelihood of consuming these farm products with any associated risk is low. However, it is essential to maintain ongoing monitoring of residual pesticides that drift onto non-target crops through aerial application. This continued monitoring aims to investigate the causes and potential risks posed by drifted pesticides, specifically to implement preventive measures against drift.

**Table 4:** Risk assessment of the pesticides that exceeded the MRL and PLS uniform standard in samples.

Pesticide	Commodity	Residue (mg/kg)	Daily food intake (g/day)	EDI^a^ (mg bw/day)	ADI (mg bw/day)	%ADI^b^ (%)
Azoxystrobin	Chili pepper	2.87	4.51	0.0129	0.200	0.0972
		2.11		0.0050	0.200	0.0715
		2.50		0.0113	0.200	0.0846
		4.85		0.0219	0.200	0.1644
Dinotefuran	Soy bean leaves	0.92	0.02	0.0000	0.100	0.0003
		2.08		0.0000	0.100	0.0006
		0.59		0.0000	0.100	0.0002
		0.22		0.0000	0.100	0.0001
Etofenprox	Peach	2.88	13	0.0375	0.08	0.7042
	White-flowered gourd	0.04	0.16	0.0000	0.08	0.0001
Propiconazole	Squash leaves	0.01	0.23	0.0000	0.10	0.0000
		0.04	0.23	0.0000	0.10	0.0001
		0.02		0.0000	0.10	0.0001
	Dureup	0.02	0.32	0.0000	0.10	0.0001
	White-flowered gourd	0.03	0.16	0.0000	0.10	0.0001
Ferimzone	Soy bean leaves	0.03	0.02	0.0000	0.019	0.0001
	Squash leaves	0.03	0.23	0.0000	0.019	0.0005
	Soy bean leaves	4.45	0.02	0.0001	0.019	0.0070
	Chili pepper	0.30	4.51	0.0013	0.019	0.1059
	Welsh onion	0.94	10.85	0.0102	0.019	0.8100
	Perilla leaves	3.35	3.06	0.0102	0.019	0.8097
	Soy bean leaves	0.39	0.02	0.0000	0.019	0.0006
	Soy bean leaves	0.88		0.0000	0.019	0.0014
	Welsh onion	0.18	10.85	0.0019	0.019	0.1536
	Chili pepper	0.47	4.51	0.0021	0.019	0.1680
		0.19		0.0008	0.019	0.0671
		0.08		0.0003	0.019	0.0271
		0.08		0.0004	0.019	0.0282
		0.01		0.0001	0.019	0.0050
	Welsh onion	0.51	10.85	0.0055	0.019	0.4342
	Chili pepper	0.21	4.51	0.0009	0.019	0.0735
	Eggplant	0.05	2.60	0.0001	0.019	0.0103
	Chili pepper	0.04	4.51	0.0002	0.019	0.0132
	Welsh onion	0.28	10.85	0.0031	0.019	0.2437
	Eggplant	0.46	2.60	0.0012	0.019	0.0946
	Tomato	0.03	14.60	0.0005	0.019	0.0381

^a^$${\text{Estimated}}\;{\text{daily}}\;{\text{intake}}\left( {{\text{mg bw}}/{\text{day}}} \right) = \,\left\{ {{\text{Residual concentration}}\left( {{\text{mg}}/{\text{kg}}} \right) \times {\text{daily food intake}}\left( {{\text{g bw}}/{\text{day}}} \right)} \right\}/{1}000$$.

^b^$$\% {\text{ADI}} = \left[ {\left\{ {{\text{EDI}}/{66}.{\text{55 kg}}\left( {\text{Average body weight of Korean}} \right)} \right\}/{\text{ADI}}} \right] \times {1}00$$.

## Conclusion

To address the labor shortage in domestic agriculture, the use of pesticides sprayed by UAVs has become necessary. However, this practice poses the risk of pesticide drift onto non-target crops and potential harm to humans. Consequently, monitoring the drift of pesticides and conducting risk analysis on non-target crops is crucial. The analysis results indicate that pesticide drift can be minimized by increasing the distance between non-target crops and the UAV spray area or by implementing windbreaks, such as planting maize, between them. Moreover, the residue of drifted pesticides tends to be lower in pulses or cereal grains, such as soybeans or maize, compared to leafy or stalk vegetables such as Welsh onions or crops with granular hairs, such as perilla leaves. Additionally, using pesticides registered for both paddy rice and other farm products in UAV spraying helps prevent unintentional pesticide contamination. Therefore, it is anticipated that these guidelines will assist farmers in avoiding violations of PLS or MRL, thereby enabling stable and continuous food production in agricultural fields and positively impacting food self-sufficiency rates. Furthermore, we envision that agricultural technologies such as UAV spraying can be utilized not only to address labor shortages but also to enhance sustainable food security.

In future research, we plan to investigate specific prevention measures for drift using a geographic information system to understand how terrain factors can potentially influence drift resulting from UAV spraying. Additionally, we will consider factors such as buffer strips, windbreaks, crop morphology, and the choice of pesticides for UAV spraying to assess their effectiveness in reducing drift.

## Supplementary Information

Supplementary Information.

## Data Availability

The datasets used and analyzed during the current study can be made available from the corresponding author upon reasonable request.
