# Supplementary material for: Monitoring and risk analysis of residual pesticides drifted by unmanned aerial spraying
Source: Sci Rep. 2023 Jul 5;13:10834. doi: 10.1038/s41598-023-36822-w (PMC10322888; doi:10.1038/s41598-023-36822-w)
Supplement: Supplementary file 1 — Supplementary Information. [file 41598_2023_36822_MOESM1_ESM.docx]

**Supplementary Material**

**Monitoring and risk analysis of residual pesticides drifted by unmanned aerial spraying**

Chang Jo Kim^1^, Xiu Yuan^1^, Min Kim^1^, Kee Sung Kyung^2,*^, Hyun Ho Noh^1,*^

^1^Residual Agrochemical Assessment Division, National Institute of Agricultural Sciences, Wanju 55365, Korea, ^2^Department of Environmental and Biological Chemistry, College of Agriculture, Life and Environment Science, Chungbuk National University, Cheongju, 28644, Korea

First author: Chang Jo Kim (C.J.K.)

*Correspondence: kskyung@cbnu.ac.kr (K.S.K.), noh1983@korea.kr (H.H.N)

Table S1. Crops collected near UAV spraying area in Boryeong, Seocheon, and Pyeongtaek.

| Crop | The number of crops | | | Total number of crops |
| --- | --- | --- | --- | --- |
|  | Boryeong | Seocheon | Pyeongtaek |  |
| Apple | 1 | 1 | 1 | 3 |
| Chili pepper | 37 | 9 | 13 | 59 |
| Cucumber | - | - | 1 | 1 |
| Dureup | 3 | - | - | 3 |
| Eggplant | 1 | - | 6 | 7 |
| Grape | - | 2 | - | 2 |
| Maize | 5 | 1 | - | 6 |
| Maize leaves | 5 | - | - | 5 |
| Peach | 1 | 5 | 2 | 8 |
| Pear | - | 3 | - | 3 |
| Perilla leaves | 1 | - | 1 | 2 |
| Sesame leaves | 1 | - | - | 1 |
| Soybean | - | 2 | - | 2 |
| Soybean leaves | 5 | 5 | 4 | 14 |
| Squash | 1 | - | - | 1 |
| Squash leaves | 4 | - | 1 | 5 |
| Tomato | - | - | 2 | 2 |
| Welsh onion | 5 | - | 5 | 10 |
| White-flowered gourd | 4 |  | - | 4 |
| White-flowered gourd leaves | 4 | - | - | 4 |

Table S2. LC–MS/MS condition for the pesticide residue analysis of azoxystrobin, chlorantraniliprole, clothianidin, dinotefuran, etofenprox, ferimzone, hexaconazole, and propiconazole in collected samples

| <LC condition> |  | | | | | | |
| --- | --- | --- | --- | --- | --- | --- | --- |
| Instrument | Nexera UHPLC, SHIMADZU, USA | | | | | | |
| Column | ACQUITY UPLC^Ⓡ^ BEH C18, 2.1 mm I.D. × 150 mm L.  (1.7 μm particle size) | | | | | | |
| Flow rate | 0.2 mL/min | | | | | | |
| Mobile phase | A: 0.1% formic acid in distilled water | | | | | | |
|  | B: 0.1% formic acid in acetonitrile | | | | | | |
|  | \| Time (min) \| A (%) \| B (%) \| \| --- \| --- \| --- \| \| 0.0 \| 90 \| 10 \| \| 1.0 \| 90 \| 10 \| \| 1.2 \| 60 \| 40 \| \| 5.5 \| 60 \| 40 \| \| 5.8 \| 10 \| 90 \| \| 11.8 \| 10 \| 90 \| \| 12.0 \| 90 \| 10 \| \| 16.0 \| 90 \| 10 \| | | | | | | |
| Injection volume | 1 μL | | | | | | |
|  |  | | | | | | |
| <Mass condition> |  | | | | | | |
| Instrument | QTRAP 5500+ system, AB SCIEX, USA | | | | | | |
| Ionspray voltage | 5500 V | | Nebulizer gas | | | 50 psi | |
| Curtain gas | 20 psi | | Drying gas | | | 50 psi | |
| Collision gas | 10 psi | | Scan type | | | MRM mode | |
| Drying gas Temp. | 500℃ | | Ion source | | | ESI+ | |
|  |  | | | | | | |
| <MRM condition> |  | | | | | | |
| Compound | Precursor ion (*m/z*) | Ion transition | | | | | |
|  |  | Quantitation ion (*m/z*) | | CE^1)^  (eV) | Confirmation ion (*m/z*) | | CE (eV) |
| Azoxystrobin | 404.1 | 372.0 | | 21 | 344.1 | | 35 |
| Chlorantraniliprole | 483.9 | 452.9 | | 27 | 285.9 | | 19 |
| Clothianidin | 250.1 | 169.0 | | 19 | 113.0 | | 39 |
| Dinotefuran | 203.1 | 129.0 | | 17 | 114.1 | | 17 |
| Etofenprox | 394.2 | 117.1 | | 21 | 359.2 | | 15 |
| (*E*)-ferimzone | 255.1 | 124.1 | | 29 | 64.9 | | 69 |
| (*Z*)-ferimzone | 255.2 | 132.1 | | 29 | 124.1 | | 29 |
| Hexaconazole | 314.0 | 70.0 | | 53 | 158.9 | | 45 |
| Propiconazole | 342.0 | 89.0 | | 101 | 123.1 | | 77 |

^1)^ Collision energy

Table S3. Recovery of pesticides sprayed by UAV in crops representing collected samples

| Pesticide | Fortification  (mg/kg) | Apple | | Wakegi onion | | Perilla leaf | | Soybean | |
| --- | --- | --- | --- | --- | --- | --- | --- | --- | --- |
|  |  | Recovery (Mean±SD, %) | RSD  (%) | Recovery (Mean±SD, %) | RSD  (%) | Recovery (Mean±SD, %) | RSD  (%) | Recovery (Mean±SD, %) | RSD  (%) |
| Azoxystrobin | 0.01 | 79.1±2.8 | 3.5 | 105.2±3.8 | 3.6 | 85.3±3.9 | 4.6 | 81.1±6.3 | 7.8 |
|  | 0.1 | 100.4±4.3 | 4.3 | 109.5±4.0 | 3.6 | 103.7±2.3 | 2.2 | 99.1±3.7 | 3.7 |
|  | 0.5 | 100.7±4.5 | 4.5 | 102.8±3.1 | 3.0 | 101.8±1.0 | 1.0 | 96.4±2.3 | 2.4 |
| Chlorantraniliprole | 0.01 | 72.5±4.9 | 6.8 | 116.6±1.3 | 1.1 | 110.5±1.6 | 1.5 | 89.8±0.7 | 0.7 |
|  | 0.1 | 93.5±1.1 | 1.2 | 112.7±5.7 | 5.1 | 99.0±1.7 | 1.7 | 107.3±5.3 | 5.0 |
|  | 0.5 | 99.5±5.5 | 5.6 | 107.7±1.4 | 1.3 | 96.8±3.1 | 3.2 | 106.2±1.7 | 1.6 |
| Clothianidin | 0.01 | 87.7±4.1 | 4.7 | 82.7±8.4 | 10.2 | 98.1±3.9 | 3.9 | 83.9±2.5 | 3.0 |
|  | 0.1 | 92.8±0.9 | 1.0 | 99.7±1.6 | 1.6 | 98.8±4.4 | 4.4 | 92.5±9.1 | 9.8 |
|  | 0.5 | 94.8±1.9 | 2.0 | 96.9±3.1 | 3.2 | 98.8±3.1 | 3.2 | 87.2±2.7 | 3.1 |
| Dinotefuran | 0.01 | 72.3±7.7 | 10.7 | 102.2±6.7 | 6.6 | 104.0±8.7 | 8.4 | 91.0±2.7 | 3.0 |
|  | 0.1 | 96.3±4.4 | 4.5 | 95.9±7.8 | 8.1 | 98.2±4.5 | 4.5 | 87.3±2.6 | 3.0 |
|  | 0.5 | 92.7±0.4 | 0.5 | 91.7±3.1 | 3.4 | 96.2±2.1 | 2.2 | 85.4±2.3 | 2.7 |
| Etofenprox | 0.01 | 96.9±3.6 | 3.7 | 81.3±4.6 | 5.7 | 93.6±4.2 | 4.5 | 81.3±8.5 | 10.4 |
|  | 0.1 | 90.9±0.7 | 0.8 | 91.8±0.2 | 0.2 | 91.7±3.1 | 3.4 | 77.1±7.1 | 9.2 |
|  | 0.5 | 91.6±7.0 | 7.6 | 85.8±2.9 | 3.4 | 95.0±5.4 | 5.7 | 73.7±3.3 | 4.5 |
| (*E*)-ferimzone | 0.01 | 96.2±4.3 | 4.5 | 82.8±4.4 | 5.3 | 88.4±3.4 | 3.8 | 79.7±3.9 | 4.9 |
|  | 0.1 | 101.9±1.7 | 1.6 | 96.5±2.5 | 2.6 | 92.2±2.7 | 2.9 | 99.5±7.6 | 7.7 |
|  | 0.5 | 102.2±1.6 | 1.5 | 91.8±2.4 | 2.7 | 91.4±5.1 | 5.6 | 100.1±3.8 | 3.8 |
| (*Z*)-ferimzone | 0.01 | 100.2±2.4 | 2.3 | 92.5±5.4 | 5.8 | 96.5±4.2 | 4.3 | 74.3±1.8 | 2.4 |
|  | 0.1 | 97.9±2.4 | 2.4 | 113.4±0.9 | 0.8 | 98.4±2.5 | 2.5 | 98.1±6.1 | 6.2 |
|  | 0.5 | 95.5±2.9 | 3.0 | 105.3±4.7 | 4.5 | 98.3±1.6 | 1.6 | 89.4±3.2 | 3.5 |
| Hexaconazole | 0.01 | 81.4±3.4 | 4.2 | 92.0±3.2 | 3.5 | 97.8±4.2 | 4.3 | 86.7±3.1 | 3.6 |
|  | 0.1 | 102.0±0.7 | 0.7 | 93.7±3.0 | 3.2 | 96.3±1.1 | 1.2 | 94.6±1.6 | 1.7 |
|  | 0.5 | 103.3±1.6 | 1.6 | 100.6±2.5 | 2.5 | 92.6±2.7 | 3.0 | 89.0±1.7 | 1.9 |
| Propiconazole | 0.01 | 99.1±3.2 | 3.2 | 96.7±5.7 | 5.9 | 94.4±3.7 | 4.0 | 101.6±2.0 | 1.9 |
|  | 0.1 | 100.9±3.0 | 2.9 | 108.9±4.8 | 4.4 | 96.4±2.6 | 2.7 | 91.4±5.4 | 5.9 |
|  | 0.5 | 104.2±1.7 | 1.7 | 101.4±1.7 | 1.7 | 95.7±1.4 | 1.5 | 84.5±2.7 | 3.2 |


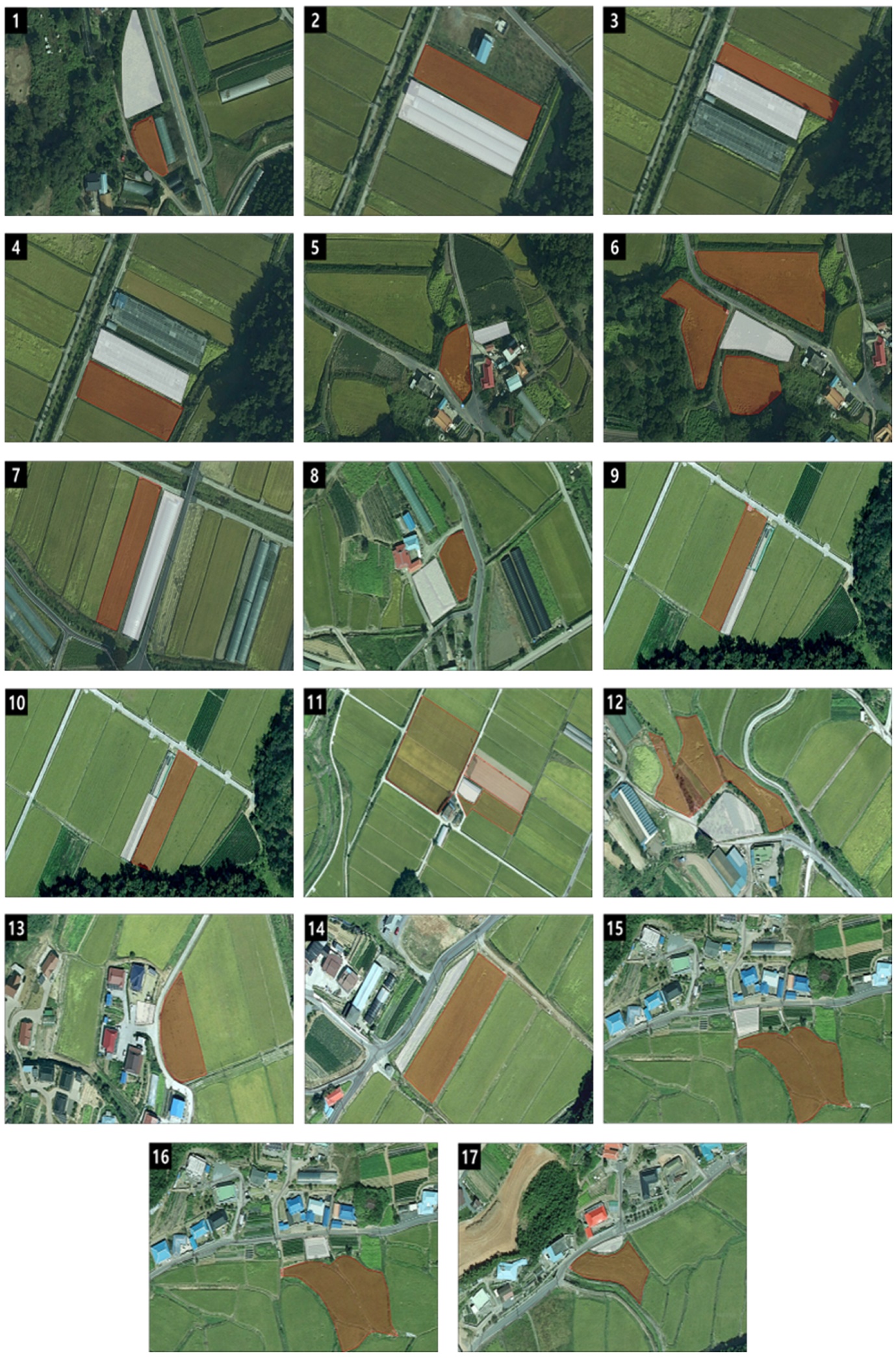


Fig. S1. Satellite images of locations in Boryeong from where the samples were collected; white indicates the locations from where the samples were collected, and red indicates the UAV-sprayed areas


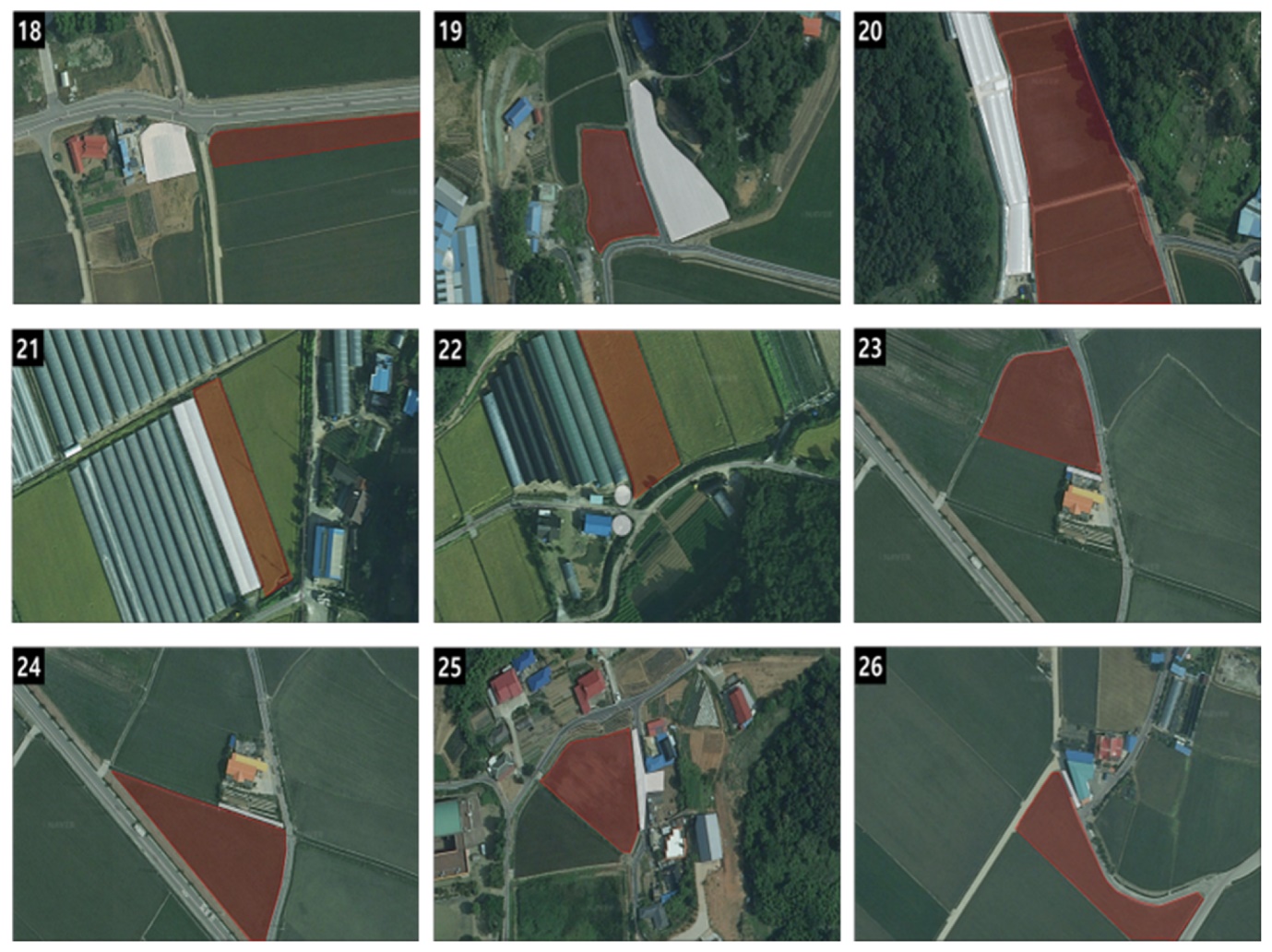


Fig. S2 Satellite images of locations in Seocheon from where the samples were collected; white indicates the locations from where the samples were collected, and red indicates the UAV-sprayed areas


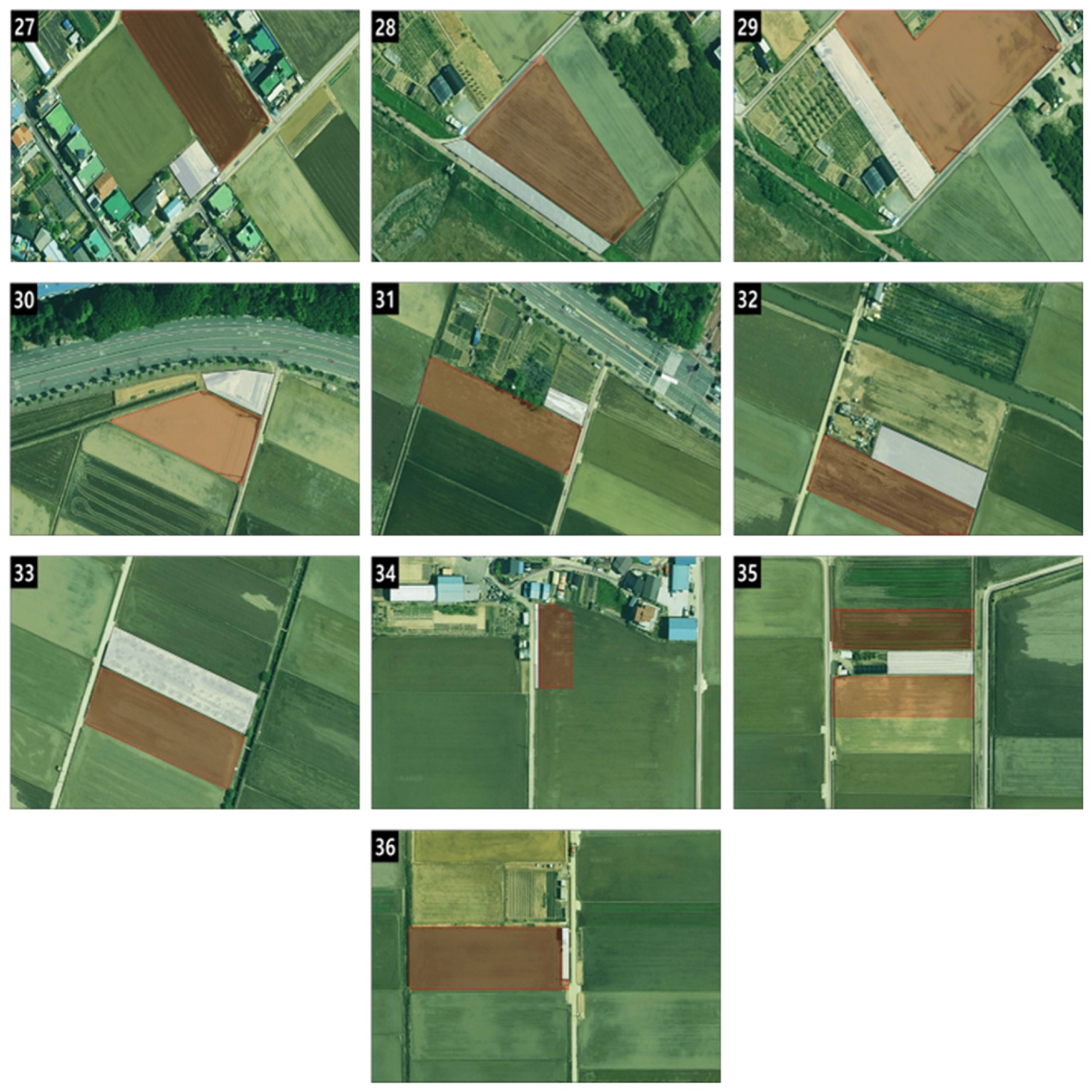


Fig. S3 Satellite images of locations in Pyeongtaek from where the samples were collected; white indicates the locations from where the samples were collected, and red indicates the UAV-sprayed areas
